# Wrist ankle acupuncture in the treatment of acute lumbar sprain: A protocol for systematic review and meta-analysis

**DOI:** 10.1097/MD.0000000000023420

**Published:** 2020-12-04

**Authors:** Shumin Liang, Guilong Zhang, Jianhong Li, Lei Zhong, Chi Zhang

**Affiliations:** a Chengdu University of Traditional Chinese Medicine; b Affifiliated Hospital of Chengdu University of Traditional Chinese Medicine; c The TCM Hospital of Longquanyi District,Chengdu, Chengdu, Sichuan, China.

**Keywords:** acute lumbar sprain, protocol, systematic review, wrist-ankle acupuncture

## Abstract

**Background:**

Acute lumbar sprain (ALS) frequently occurs in the young and middle-aged people, causing great harm to people's quality of life. The systematic review program was designed to describe a meta-analysis to evaluate the efficacy and safety of wrist-ankle acupuncture (WAA) in treating patients with ALS.

**Methods and analysis:**

Our systematic review will search electronically and manually for WAA treatments for ALS by August, 2020, regardless of publication status and language. Databases include: MEDLINE, PubMed, EMBASE, Springer, Web of Science, Cochrane Library, WHO International Clinical Trial Registration Platform (ICTRP), Chinese Medicine Database (TCMD), China National Knowledge Infrastructure (CNKI), Chinese Biomedical Literature Database (CBM), China Science Journal Database (VIP), and Wanfang Database. Other sources of information, including bibliographies and meeting minutes for identified publications, will also be searched. Manually search for grey literature, including unpublished conference articles. Any clinical randomized controlled trials related to WAA treatments for ALS, regardless of publication status and language limitations, will be included in the study. Study selection, data extraction, and research quality assessment will be done independently by 2 researchers. The primary outcome included the effective rate and visual analogue scale (VAS) or other validated scales used to relieve pain after the treatment. If possible, meta-analysis will be performed using RevMan V.5.3 statistical software. If it is not suitable for meta-analysis, a descriptive analysis or subgroup analysis is performed.

**Results:**

This study will provide a comprehensive review and evaluation of the available evidence for the treatment of ALS using WAA.

**Conclusion:**

This study will provide new evidence to evaluate the effectiveness and side effects of WAA on ALS. Because the data is not personalized, no formal ethical approval is required.

**Registration number:**

PROSPERO CRD42020162945

## 1 Introduction

### 1.1 Description of the condition

Acute lumbar sprain is an acute injury of the lumbar soft tissue including ligaments, muscles, or facet joints by an inappropriate lumbar activity. According to the etiology, pathogenesis, location and relevant clinical manifestations of waist sprain, modern physicians classify waist sprain into the categories of “tendon injury”, “falling injury”, “blood stasis and back pain” and “arthralgia syndrome”. But for the main symptom of acute lumbar sprain - low back pain, doctors of all dynasties have analyzed and discussed the cause and classification of the disease. Modern medicine defines acute lumbar sprain as soft tissue disease caused by acute injury of lumbar tissue due to sudden force or excessive weight, which lasts less than 6 weeks. Major injuries to the erector spinal muscle and its fascia, the supraspinous ligament and the primordial tendon are also involved.

Although Chinese and Western medicine have studied etiology of acute lumbar sprain based on different thinking systems and observation models, the results are very similar, which can only be attributed to internal and external causes. The internal causes are age, constitution, occupation or lifestyle, and anatomical structure. The external cause is falling injury, feeling cold and so on. In addition, Western medicine pays more attention to the microscopic level of etiology and puts forward the theory of gate control,^[1]^ the theory of dynamic balance disorder ^[2]^ aseptic inflammation theory, the theory of soft tissue anatomical location shift.^[3]^

Patients with acute lumbar sprain, local pain, fixed position, pressure pain, muscle contact spasm. Patients with limited movement, posture or lateral curvature, mainly due to spasm of the erector spine side of the waist, this pathological position is mainly to protect the spine. Back muscles are tense and are more pronounced in acute injuries. All ALS investigated in this study were based on patients with such manifestations.

At present, there are various treatments for ALS. Modern medicine mostly uses western medicine, blocking therapy, immobilization therapy and physical therapy. Among them, fixed braking is the most commonly used method in clinical practice, which is of great significance to improve the lower back pain, function and patients’ quality of life. More than 80% of patients with acute lumbar sprain can relieve pain after 2 weeks of bed rest.^[4]^ Acute pain relief can be combined with rehabilitation training, usually start the active contraction of muscles under the state of rest, if there is no obvious pain, you can continue to increase the amount of exercise, continuous training to enhance muscle strength, stabilize the spine, so as to reduce the load of the lumbar spine, to protect the lumbar structure, prevent recurrence of the purpose.

Western medicine treatment mainly includes internal medicine and local medicine. Internal medicine for analgesics, divided into 2 categories. One is anti-inflammatory and antipyretic, which blocks cyclocytes and inhibits prostaglandin synthesis to treat mild pain, such as non-steroidal anti-inflammatory drugs. The other ACTS on the central nervous system, selectively relieving or eliminating pain and treating various forms of acute pain, such as morphine. Local use medicine basically is divided into local close, hot compress, stick apply, besmear with wine or oil. Blocking therapy is also a kind of drug therapy, which uses local anesthetics and hormones to inject in the local pain site, so as to achieve the closure of pain points and block spinal nerves.^[5]^

Physical therapy refers to the use of natural or artificial physical factors to prevent and treat diseases, belongs to the external stimulation, in the regulation of human physiology, enhance the ability of adaptation and promote functional recovery has the dual role of information and dynamic, especially for soft tissue injury. There are mainly electric therapy, ultraviolet ray, infrared ray and so on. In recent years, physical therapy has been paid more and more attention. This method has obvious effect in anti-inflammatory and analgesic, relieving spasm, increasing muscle strength and improving lumbar function.^[6]^

The method of TCM treatment of acute lumbar sprain is diversiform, curative effect is distinct, consider etiology mainly dropped waist pain, the more belongs to qi and blood stasis, treatment is given priority to with qi, activating blood, treatment methods mainly include traditional Chinese medicine, acupuncture, massage, bloodletting therapy, such as relative to the modern therapy, its advantage lies in low price, curative effect is outstanding, the pain quickly, etc, among them with acupuncture analgesia effect is most obvious. Carpal and ankle acupuncture is a kind of acupuncture, which is especially effective in treating ALS.

In recent years, there have been increasing reports on the treatment of ALS with wrist and ankle acupuncture, but no systematic review by scholars has been found in literature review. Therefore, this study takes WAA treatment of ALS as the research objective, and summarizes and analyzes relevant randomized controlled trial (RCT) experimental research reports before August 2020 to evaluate its effectiveness and safety.

### 1.2 Description of the intervention

Wrist-ankle acupuncture (WAA) is a kind of acupuncture therapy initiated by Professor Zhang Xinshu, and is one of the traditional Chinese acupuncture and moxibustion characteristic diagnosis and treatment items. It is named because it only performs subcutaneous acupuncture on the wrist and part of the limbs to treat diseases.^[7]^ Carpal and ankle acupuncture therapy is to summarize the parts of the symptoms and symptoms in the body on both sides of the 6 longitudinal areas, in both sides of the wrist and ankle set 6 points into the needle, diaphragm as the boundary, according to the selected points for treatment.

### 1.3 How might intervention work

Wrist and ankle acupuncture has functions of dredge meridians and collaterals, harmonizing viscera and so on. It is suitable for various pain syndromes and diseases of viscera. To sum up, areas 4, 5, and 6 (including area 1) under the wrist and ankle needle are connected with the waist through and through the air. This stimulation area (point) is the generalization of the meridian of foot sun and meridian; during the treatment of wrist and ankle acupuncture, the active and passive activities of the patients can promote the recovery of the injured and displaced muscles and ligaments.^[8]^

Modern medical scholars believe that the immediate analgesic effect of wrist-ankle acupuncture is attributed to neuromodulation, while the permanent effect cannot be excluded from the participation of humoral factors.^[9]^ Acupuncture afferent information can activate many endogenous opioid peptides, such as enkephalin and strong enkephalin, thus inhibiting the transmission of hurtful information. At the same time, it can also promote the synthesis and release of many monoamine transmitters (5-hydroxytryptamine, dopamine, etc), and various transmitters interact to participate in acupuncture analgesia process together. The results showed that the acupoints of electroacupuncture could improve the pain threshold and the content of acetylcholine in the cortex was also increased. Acupuncture has produced significant increases in the blood of the brain's intracytoids, but no histamines.^[10]^ Acupuncture treatment in 4, 5 and 6 areas (including 1 area) may have the same or similar mechanism.

### 1.4 Objectives

To assess the efficacy and safety of wrist-ankle acupunture in treating acute lumbar sprain.

## 2 Methods and analysis

### 2.1 Study registration

PROSPERO registration number is RD42020162945. This protocol report is structured according to the preferred reporting items for systematic reviews and meta-analyses protocols (PRISMA-P) statement guidelines.^[11]^

The review will be conducted in accordance with the PRISMA guidelines and follows the recommendations of the Cochrane Handbook for Systematic Reviews of Interventions.^[12,13]^

If we refine the procedures described in this protocol, we will update the record in the PROSPERO and disclose them in future publications related to this study.

### 2.2 Inclusion criteria for study selection

#### 2.2.1 Types of studies

We will include in the RCTs without the limitations of language and publicity. However, animal mechanism studies, case reports, self-controlled, non-randomized controlled trials, random crossover studies, and quasi-randomized trials will be excluded.

#### 2.2.2 Types of participants

Regardless of gender, age, ethnicity, education, and economic status, patients with ALS who meet the following diagnostic criteria (e.g.,《diagnostic and curative effect criteria of TCM diseases and syndromes》(promulgated by the National Administration of Traditional Chinese Medicine))^[14]^

#### 2.2.3 Types of interventions

WAA refers to a method of stimulating special acupuncture points which in wrist or ankle. Other methods such as acupressure, moxibustion, normal acupuncture, laser acupuncture, drug acupuncture, dry needle or transcutaneous electrical nerve stimulation will be excluded. Sham WAA includes acupuncture at other part of body besides wrist or ankle, inappropriate acupoints of WAA, sham acupuncture at acupoints, sham acupuncture at non-acupoints, non-piercing acupuncture and sham intervention).^[15]^

Complete the following comparison.

1. WAA compared with no treatment.
2. WAA compared with placebo or sham acupuncture.
3. WAA compared with other active therapies.
4. WAA in addition to active therapy compared with the same active therapy.

We will assess and compare the WAA according to how the acupuncturists have been trained and educated, on their clinical experience, on total numbers of WAA sessions, and on the treatment duration and frequency, and so on.^[16]^

#### 2.2.4 Types of outcome measures

The primary outcome was the visual analog scale, the brief pain inventory-short form, or other validated scales used to improve ALS after treatment.^[17,18]^ Secondary outcomes include response rate, Oswestry disability index, self-rating anxiety scale, self-rating depression scale, Pittsburgh sleep quality index, recurrence rate during the follow-up period, and adverse events.^[19–21]^ The system review will be performed independently.

### 2.3 Search methods for identification of studies

#### 2.3.1 Electronic searches

Our systematic review will electronically and manually search for all RCTs for acupuncture treatment of CP, regardless of publication status and language, by September 2019. Databases include: MEDLINE, PubMed, EMBASE, Springer, Web of Science, Cochrane Library, WHO International Clinical Trials Registry Platform (ICTRP), Traditional Chinese Medicine databases (TCMD), China National Knowledge Infrastructure (CNKI), China Biomedical Literature Database (CBM), Chinese Scientific Journal Database (VIP) and Wan-Fang database. The following search terms will be used: wrist-ankle acupuncture or wrist ankle acupuncture or carpus-ankle acupuncture or wrist and ankle acupuncture and acute lumbar sprain or acute waist sprain or acute sprain waist or acute lumbar injury or “acute waist wrench or acute lumbar strain. Use the same search term in the Chinese database. Develop a search strategy based on the Cochrane Handbook guidelines,^[22]^ PubMed's search strategy is shown in Table 1.

**Table 1:** PubMed search strategy.

Number	Search terms
1	Randomised controlled trial
2	Controlled clinical trial
3	Randomised
4	Randomly
5	Placebo
6	Trial
7	Groups
8	1 or 2–7
9	Wrist-ankle acupuncture
10	Wrist ankle acupuncture
11	Carpus-ankle acupuncture
12	Wrist and ankle acupuncture
13	9 or 10–12
14	Acute lumbar sprain
15	Acute waist sprain
16	Acute sprain waist
17	Acute lumbar injury
18	Acute waist wrench
19	Acute lumbar strain
20	14 or 15–19
21	8 and 13
22	20 and 21

#### 2.3.2 Searching other resources

The reference list of the confirmed publications will be scanned by us for more trials. We will search PubMed, Turning Research into Practice (TRIP) database and the Cochrane Library for existing systematic reviews possibly relevant to this systematic review to search their reference lists for additional trials. We will also search for meeting minutes related to this topic. Manually search for grey literature, including unpublished conference articles.

### 2.4 Data collection and analysis

#### 2.4.1 Selection of studies

**Figure 1:**
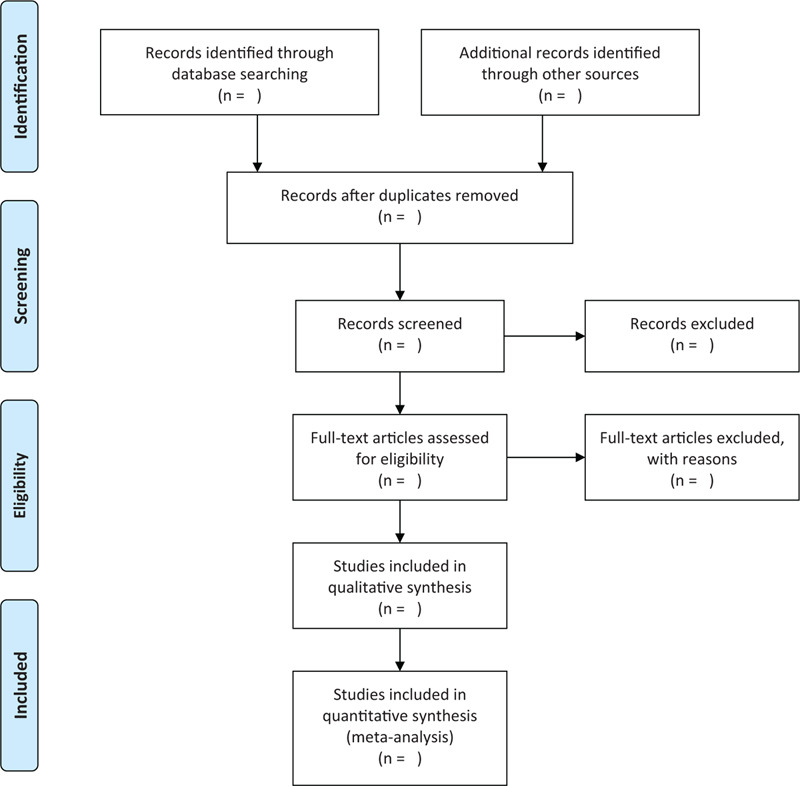
Flow diagram of studies identified.

We plan to conduct a review of this system between October 1, 2019, and July 30, 2020. All reviewers are trained to begin the review to ensure that the background and purpose of the review are fully known. After the electronic retrieval, all the records will be uploaded to the database created by EndNote software (V.X8). Records that are selected manually or from other sources will also be moved to the same database. The 2 review authors (SL and ZL) will independently screen the titles, abstracts, and keywords of all retrieved trials and determine which trials meet the inclusion criteria we have developed. We will obtain the full text of all relevant trials for further review and evaluation. Excluded trials will be recorded and explained. Any dispute will be resolved by the 2 authors (SL and ZL) and the third author (CZ), and arbitration if necessary. If necessary, we will contact the trial author for clarification. The primary selection process is shown in a PRISMA flow chart (Fig. 1).

#### 2.4.2 Data extraction and management

Each author will extract data independently from the selected report or study and fill out a data extraction form. We will obtain data on general information, participants, methods, interventions, outcomes, adverse events, conflicts of interest, ethical perceptions, and other information. When the reported data is insufficient, we will contact the author for more information. Any disagreement will be resolved by discussion between the 2 authors, and any further disagreement will be arbitrated by the third author (CZ).

#### 2.4.3 Assessment of risk of bias in included studies

The authors (JL and ZL) will use the Cochrane collaboration's bias risk assessment tool to assess the risk of bias for all included studies. We will assess the risk of deviation in sequence generation, allocation sequence hiding, the blindness of participants and staff, result evaluators, incomplete result data, selective result reporting, and other sources of deviation. This review uses L, U, and H as key to these assessments, where L (low) indicates a lower risk of bias, U (unclear) indicates an uncertain risk of bias and H (high) indicates a higher risk of bias. If inconsistent results occur, the final decision will be made by the third author (CZ). The information contained in the study on the biased assessment of risk was summarized in tabular form and the results and impacts were critically discussed. If the information is not clear, we will try to contact the author. For duplicate articles, we select only the original.

#### 2.4.4 Measures of treatment effect

Data analysis and quantitative data synthesis will be performed using RevMan V.5.3. For continuous data, if there is no heterogeneity, we will use mean difference (MD) or standard MD (SMD) to measure the therapeutic effect of 95% CIs. If significant heterogeneity is found, a random effects model will be used. For the 2-category data, we will use the 95% CIs risk ratio (RR) for analysis.

#### 2.4.5 Unit of analysis issues

We will include a meta-analysis of data from parallel group design studies. Only the first phase of the data will be included in the random crossover trial. In these trials, participants were randomly assigned to two intervention groups individually, and individual measurements for each outcome of each participant were collected and analyzed.

#### 2.4.6 Dealing with missing data

We will attempt to contact the first author or correspondent author of the study to request missing or insufficient data. If possible, an intent analysis (including data from all participants) will be performed and a sensitivity analysis will be used to determine if the results are inconsistent.

#### 2.4.7 Assessment of heterogeneity

We will use the I^2^ statistic to quantify the inconsistency. When the I^2^ value is less than 50%, the study will not consider having heterogeneity. When the I^2^ value exceeded 50%, there was significant statistical heterogeneity between trials without meta-analysis. A subgroup analysis will be conducted to explore possible causes.

#### 2.4.8 Assessment of reporting biases

The funnel plot is used to detect reported bias and the effects of small-scale studies. If there are more than 10 studies, we will use the Egger method to test the asymmetry of the funnel plot.^[23]^ All eligible trials, regardless of the quality of their methods, will be included.

#### 2.4.9 Data synthesis

When the meta-analysis is performed, RevMan V.5.3 will be used for data synthesis. The result will be expressed as the RR value of the binary data and the SMD value of the continuous data. If the I^2^ test is less than 50%, a fixed effect model is used for data synthesis. If the I^2^ test is between 50% and 75%, a random effects model is used for data synthesis. If the I^2^ test is higher than 75%, we will investigate the possible causes from a clinical and methodological perspective and provide a descriptive analysis or a subgroup analysis.

#### 2.4.10 Subgroup analysis

There is no presubgroup plan. Subgroup analysis was performed based on control interventions and different outcomes.

#### 2.4.11 Sensitivity analysis

Sensitivity analysis is the robustness of the main decisions in the test review process. Includes the impact of methodological quality, sample size, and missing data. Meta-analyses will be repeated and low-quality studies will be excluded. The results will be compared and discussed based on the results.

#### 2.4.12 Grading the quality of evidence

The quality of evidence for all results will be judged by the method of recommending the grading, assessment, development, and evaluation of the working group. The following factors will be assessed: bias, consistency, directness, accuracy, publication bias, and risk of attachment points. The assessment will be divided into 4 levels: high, medium, low, or very low.

#### 2.4.13 Ethics and dissemination

Because we are not directly targeting individuals, and extracting data without privacy, formal ethical approval is not necessary. The results of the study will be disseminated through peer-reviewed publications or conference reports.

## 3 Discussion

Acute lumbar sprain is a common but refractory condition. Up to date, there are no official treatment guidelines to follow. Consequently, numerous studies have been focused on alternative therapies, because currently Western medicine does not have much to offer except anti-inflammatory analgesics. Today more and more patients turn to acupuncture, including WAA, EA.

However, WAA response varies greatly from patient to patient and different conclusions have been drawn in different clinical trials. To address inconsistency between clinical trials, provide more clinical evidence and thus set up a guideline or treatment protocol for acute lumbar sprain, we have conducted this systematic review and meta-analysis for the first time.The evaluation will consist of 4 parts: identification, inclusion studies, data extraction, and data synthesis. We hope that this systematic review will provide clinicians with more convincing evidence in the decision-making process for the treatment of ALS.

## Author contributions

**Conceptualization:** Shumin Liang.

**Methodology:** Guilong Zhang.

**Software:** Jianhong Li.

**Supervision:** Lei Zhong.

**Validation:** Chi Zhang.

**Writing – original draft:** Shumin Liang, Guilong Zhang, Chi Zhang.
